# Hydrological disasters and health losses: an index-based analysis for Brazil

**DOI:** 10.11606/s1518-8787.2026060007333

**Published:** 2026-07-06

**Authors:** Gisele Paixão Pereira, José Firmino de Sousa, Thomás Rocha Ferreira, Paulo Victor Maciel da Costa, Andrêa Jacqueline Forte Ferreira, Julia Moreira Pescarini, Joanna Miguez Nery Guimarães, Sofia Rafaela Maito Velasco, Danielson Jorge Delgado Neves, Jean Souza dos Reis, Kecia Cristina Miranda da Silva, Gervásio Ferreira dos Santos, Rita de Cássia Ribeiro Silva, Roberto Fernandes Silva Andrade, Maurício Lima Barreto

**Affiliations:** 1 Fundação Oswaldo Cruz. Centro de Integração de Dados e Conhecimentos para Saúde. Salvador, BA, Brasil; 2 Universidade Federal da Bahia. Faculdade de Economia. Departamento de Economia. Salvador, BA, Brasil; 3 Universidade Federal de Alagoas. Instituto de Ciências Astronômicas. Maceió, AL, Brasil; 4 London School of Hygiene and Tropical Medicine. Faculty of Epidemiology and Population Health. London, United Kingdom; 5 Instituto Nacional de Pesquisas Espaciais. Cachoeira Paulista, SP, Brasil

**Keywords:** Floods, Public Health, Health Facilities, Vulnerability Analysis, Health Inequalities

## Abstract

**OBJECTIVE:**

To develop and apply composite indices to assess immediate losses caused by hydrological disasters in Brazil between 2000 and 2023, with a focus on human losses and damages to health infrastructure.

**METHODS:**

Data were obtained from the Integrated Disaster Information System, which consolidates national records of hydrological disasters. Seven indicators were selected for the Human Loss Index and four for the Health Infrastructure Loss Index. Indicators were normalized using Min-Max procedures and weighted through a combined Analytic Hierarchy Process and entropy method. The General Severity Index was calculated as the arithmetic mean of Human Loss Index and Health Infrastructure Loss Index, stratified into four levels of severity. Spatial and temporal analyses were conducted to identify critical regions and municipalities.

**RESULTS:**

The findings reveal significant heterogeneity in disaster impacts across time and space. Human losses exhibited greater variability than health infrastructure losses, with notable peaks in 2006, 2011, 2015, and 2019. Infrastructure damages were less frequent but highly disruptive, especially in 2012, 2019, and 2023. Municipalities in the Amazon region, particularly Japurá and Atalaia do Norte, registered the highest combined indices, reflecting compounded vulnerabilities from geographic isolation, fragile infrastructure, and socioeconomic inequalities. High General Severity Index scores were concentrated in the North, while human losses were more widely distributed, including along the densely populated southeastern coast. Results confirm that small and socioeconomically vulnerable municipalities are disproportionately affected, with limited institutional capacity to respond.

**CONCLUSIONS:**

Hydrological disasters in Brazil are strongly mediated by social inequalities, deficient urban planning, and weak preventive policies. Human and infrastructure losses disproportionately affect vulnerable populations, particularly in the North, where access to health services is precarious. The findings emphasize the need for territorially integrated strategies that combine climate adaptation, poverty reduction, and investments in resilient public health infrastructure.

## INTRODUCTION

Climate change intensified by human activity is reshaping the global landscape of disaster risk by amplifying the intensity of hazards and the conditions that foster their occurrence.

Key concerns include: (i) the increasing frequency and intensity of extreme weather events; (ii) changes in hydrological cycles; (iii) sea level rise and heightened coastal vulnerability; and (iv) the emergence of new risks and the shifting geographical distribution of existing ones^1-6^.

These changes have severe societal impacts, leading to damage to human health and substantial economic losses. It is estimated that direct health costs will reach between 2 and 4 billion annually by 2030, with an additional 1.1 trillion in health system expenses by 2050, alongside potential global economic losses of 12.5 trillion in the same period^7^. Such figures highlight both the urgency of effective climate action and the need for adaptive strategies that account for evolving risk projections.

In response, in 2015, the United Nations member states adopted the Sendai Framework for Disaster Risk Reduction, committing to improving disaster risk governance by 2030. That same year, the Sustainable Development Goals (SDGs) were also established, with clear targets to reduce mortality, the number of people affected, and the direct economic losses from disasters. To achieve these goals, member states are required to monitor and systematically report losses, as reliable data are essential for measuring and tracking international targets^8,9^. However, only a few countries maintain comprehensive databases on human and economic losses, and disaster severity is often assessed solely by the magnitude of damages, without considering factors such as population density^10^.

Previous studies have examined the impacts of natural disasters on health systems and society in Brazil. Minervino and Duarte^11^ analyzed data from national and international information systems to assess material damage to public health services and society resulting specifically from floods and flash floods between 2010 and 2014, highlighting the magnitude and regional heterogeneity of hydrological disasters. Freitas et al.^12^ assessed the costs of natural disasters to health establishments in Brazil between 2000 and 2015, allowing for comparisons between hydrological events and other disaster types. While these studies provide important evidence on material damage and economic costs, they do not propose a synthetic measure that captures human losses and damage to health infrastructure in a comparable way across municipalities.

Against this backdrop, the construction of indices becomes a strategic tool for governance, as it enables disaster assistance, recovery and reconstruction programmes; the assessment of future risks; the evaluation of the economic feasibility of preventive investments; the monitoring of impact patterns and trends; and thematic analyses aligned with international commitments^9^.

The aim of this study is to assess human losses and damage to health infrastructure caused by hydrological disasters in Brazil by developing an immediate loss index that accounts for population size, thereby enabling comparability across contexts. Data were drawn from the Integrated Disaster Information System (S2ID), which records occurrences across all Brazilian municipalities from 1991 to 2023, as well as emergency and calamity decrees. The index was constructed using weighting methods based on the Analytic Hierarchy Process (AHP) and the entropy method, following the approaches proposed by recent literature^10,13^.

The analysis is justified on three main grounds. First, hydrological disasters represent the leading acute climate-induced mortality risk^7^. This category includes floods, heavy rainfall, flash floods, and landslides^14^. Within this context, according to the international EM-DAT database^15^, Brazil ranked first in 2023 with the highest number of recorded floods^12^. EM-DAT records that, between 2021 and 2023, Brazil registered 31 flood-related disasters, resulting in 714 deaths and affecting more than 2.1 million people.

Second, in low- and middle-income countries such as Brazil, the growing frequency and intensity of disasters pose serious challenges to public health systems. Socioeconomic inequalities, as reflected in housing conditions, health status, and access to services, combined with institutional constraints, exacerbate existing vulnerabilities. Furthermore, these countries often lack the institutional capacity to record disaster-related losses systematically and generally do not have consistent historical data^9^. Finally, Brazil's continental dimensions and pronounced regional heterogeneity make it a particularly relevant case study for research on disaster losses. The availability of a national database further strengthens its significance.

This study contributes to the literature by proposing, to the best of our knowledge, the first index of losses from hydrological disasters in Brazil. The work adapts the model developed by Zhao et al.^10^, originally applied to Shanghai, by incorporating variables on health infrastructure losses, an aspect of critical importance in the Brazilian context, where more than 70% of the population relies on the public health system^16^. The loss of health infrastructure can severely compromise access to essential healthcare.

The findings are expected to strengthen the robustness of the existing evidence base and inform more effective disaster risk reduction policies in Brazil.

## METHODS

### Data

The use of indicators and composite indices is not an end in itself, but rather a tool to support systematic monitoring, progress evaluation, and disaster risk management. In this regard, the Monitoring Sendai Framework^a^ provides an internationally recognized reference, establishing a set of indicators designed to track progress toward the seven global targets of the Sendai Framework for Disaster Risk Reduction and its related dimensions within the SDGs, particularly SDGs 1, 11, and 13. Although the indicators proposed in this study are derived from S2ID, the construction of the Human Loss Index, the Health Infrastructure Loss Index, and the General Severity Index is conceptually aligned with the Sendai Framework's monitoring logic, as it focuses on human impacts, damage to critical infrastructure, and the generation of comparable metrics to inform risk governance.

This study proposes an approach to assess losses from hydrological disasters across municipalities by analyzing and comparing recorded occurrences in Brazil along both temporal and spatial dimensions. The dataset used in this study originates from the S2ID, which consolidates various tools and data products from *Secretaria Nacional de Proteção e Defesa Civil* (Brazil's National Secretariat for Civil Protection and Defense), to enhance the quality and transparency of disaster and risk management in the country. Through S2ID, one can access the Digital Atlas of Disasters in Brazil, which catalogs disaster notifications that occurred in the country between 1991 and 2012. However, data up to 2012 were derived from digitized paper-based disaster protocols recorded during that period. Following the formalization of disaster reporting via S2ID in 2013, the data from this point onward are extracted from official records submitted by local civil defense agencies or municipal governments through the S2ID platform^17^.

The *Formulário de Informações do Desastre* (Disaster Information Form) is the standardized reporting document and is structured into eight sections:

1. Municipality identification and socioeconomic context;
2. Disaster classification;
3. Date and time of occurrence;
4. Characteristics of the affected area;
5. Disaster causes and impacts;
6. Human, material, and environmental damages;
7. Assessment of public and private economic losses; and
8. Identification of the reporting institution^17^.

Although the official data collection process was initially designed primarily to support resource requests for emergency response and reconstruction efforts, its standardization and systematic processing led to the creation of a comprehensive national disaster database that can serve as a strategic management tool.

However, despite being the main and most comprehensive official data source on disasters in Brazil, the Digital Atlas of Disasters has important methodological limitations that must be acknowledged to enable a more critical and accurate interpretation of its results. First, the completion of the Disaster Information Form depends on self-reporting by local civil defense units or other governance bodies. This introduces potential political bias, as in some cases the declaration of a state of emergency may be used to expedite access to financial resources, regardless of the actual severity of the event^18^.

Moreover, the system primarily records the situation at the time of the initial disaster notification, without subsequent updates that would reflect the evolving conditions of affected populations, material damages, or economic losses. This gap can lead to a significant underestimation of the true impacts of the events analyzed^18,19^. Additionally, data classification is based on the municipality rather than the disaster event itself, complicating the analysis of regionally extensive disasters, such as floods and flash floods that cross municipal or even state boundaries. There are also coverage gaps in remote or less developed areas, where institutional capacity for disaster reporting is limited^19^. Despite these limitations, the use of the Atlas remains valid and relevant, as it has already been validated by the existing literature, especially given the absence of alternative databases with equivalent spatial granularity and temporal coverage^20,21^.

We selected seven loss indicators to construct the Human Loss Index (HLI): fatalities, injured people, illnesses, displaced people, evacuees, missing people, and others affected. Four indicators were selected to develop the Health Infrastructure Loss Index (HII): destroyed health facilities, damaged health facilities, the monetary value of affected health facilities, and public expenditures for medical assistance and emergency response. The HLI is intended to characterize the magnitude of human losses caused by hydrologic disasters, while the HII assesses the impacts on critical healthcare infrastructure that supports communities or society. To allow comparability across municipalities with different population sizes, the indicators were adjusted to a base of 100 thousand inhabitants. The Chart provides detailed information about the selected indicators.

**Chart:** Indicators used in the construction of Human Loss and Health Infrastructure Loss Indexes.

Index	Indicator	Description
Human Losses	Fatalities	People who lost their lives as a direct result of the effects of the disaster (Units)
	Injured people	People who suffered injuries as a direct result of the effects of the disaster and require medical and hospital intervention, health materials, and supplies (Units)
	Illnesses	People who developed pathological processes as a direct result of the effects of the disaster (Units)
	Displaced people	People in need of public shelter, such as temporary housing, due to damage or threat of damage caused as a direct result of the effects of the disaster (Units)
	Evacuees	People who have center their homes but do not require public shelter (Units)
	Missing people	People who need to be found because, as a direct result of the effects of the disaster, they are at risk of imminent death and in unsafe/dangerous places (Units)
	Others affected	People directly affected by the disaster (excluding those already reported) (Units)
Health Infrastructure Losses	Destroyed health facilities	Public health facilities destroyed (Units)
	Damaged health facilities	Damaged public health facilities (Units)
	Monetary value of health facilities affected	Value of material damage to public health facilities, adjusted to December 2023 values (R$)
	Public expenditures related to medical assistance and emergency response	Public losses with medical assistance, public health, and medical emergency care, adjusted to December 2023 values (R$)

Although the database includes records of emergency and disaster events since 1991, monetary data at constant prices is available only from 1995 onwards, due to the currency change in Brazil in 1994. Furthermore, data on the indicators for destroyed health infrastructure and public expenditure for medical assistance and emergency response were not recorded before 2000. For these reasons, our analysis spans the period from 2000 to 2023. It should also be noted that the two aforementioned variables lack data for 2002 and 2006; therefore, the annual General Severity Index (GSI) does not include these indicators as components for those specific years. For the health infrastructure index, we chose not to report values for these two years.

### Empirical Strategy

Initially, the indicators were processed using the min-max normalization method. Normalization is relevant because, whenever indicators within a dataset are incommensurable or expressed in different units of measurement, it becomes necessary to convert these indicators into a comparable scale^10,22,23^. The normalization formula is as follows:


$$X^{*}=\frac{X-X_{min}}{X_{max}-X_{min}}$$

where X* represents the normalized indicator value, *X* is the original indicator value, and *X_max_* and *X_min_* correspond, respectively, to the maximum and minimum values recorded for each indicator between 2000 and 2023. Subsequently, the weights of the indicators are calculated based on their relative importance using a combination of the Analytic Hierarchy Process (AHP) and the entropy method, enhancing the robustness of the weight calculation^10,13^. The AHP is a structured subjective approach for multicriteria decision-making that employs pairwise comparisons to assign relative weights to criteria and alternatives within a hierarchical framework. Based on Saaty's scale, AHP converts qualitative judgments into quantitative values, synthesizing priorities and verifying the consistency of evaluations^24^. It involves structuring the problem into hierarchical levels, systematically comparing elements, and computing weights via eigenvalues.

Entropy, in contrast, is an objective weighting method, a statistical technique used to determine weights in composite indices, ensuring that the most informative variables exert a greater influence on the final index^13^. Entropy measures the uncertainty or dispersion of a variable. The calculation of entropy is expressed as follows:


$$H_{j}=-k\overset{n}{\underset{i=1}{\sum }}p_{ij}\ln p_{ij}$$

where *H*_*j*_ represents the entropy of the normalized indicator j; *p*_*ij*_ is the proportion of the value of indicator j for item *i, k* is a normalization constant defined as (*K*=1/ln (*n*)) (where *n* is the number of observations). Subsequently, the degree of diversification (*d*_*j*_), is calculated as follows:


$$d_{j}=1-H_{j}$$

Finally, the entropy-based relative weight for each indicator is calculated as follows:


$$w_{j}=\frac{d_{j}}{\sum d_{j}}$$

The combined weights, designed to enhance calculation robustness, are obtained through the following formula:


$$w=\frac{w_{j}^{a}w_{j}^{b}}{\sum_{j=1}^{n}w_{j}^{a}w_{j}^{b}}$$

where *w* represents the combined weight, $w_{j}^{a}$ corresponds to the weight obtained through AHP, and $w_{j}^{b}$ is the weight derived from the entropy method. After completing the normalization and determining the weights of the indicators, the HLI and the HII can be calculated using a weighted average, as follows:


$$c_{i}=\overset{n}{\underset{j=1}{\sum }}w_{j}x_{ij}$$

where *C*_*i*_ represents the index value for item i; *w*_*j*_ is the weight assigned to the j-th indicator (HLI or HII); and *x*_*ij*_ is the normalized value of the j-th indicator. The GSI, in turn, is calculated as the arithmetic mean of the two indices, as shown below:


$$GSI_{i}=\frac{HLI_{i}+HII_{i}}{2}$$

The equal-interval method was applied to classify the indices, a widely used approach in disaster risk assessment studies^10^. The indices were stratified into four classification levels: Level 1, corresponding to the lowest values (0 to 0.25); Level 2, encompassing the lower-intermediate range (0.25 to 0.50); Level 3, referring to the upper-intermediate range (0.50 to 0.75); and Level 4, representing the highest values (0.75 to 1), which denote municipalities with the greatest magnitudes of losses.

## RESULTS

**Figure 1:**
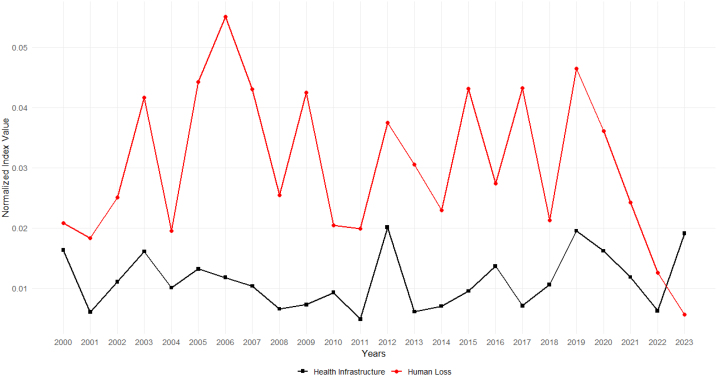
Human and health infrastructure losses caused by hydrological disasters over time.

Figure 1 shows that damage variability over time is greater for human losses than for health infrastructure losses. The HLI follows a relatively recurrent pattern and shows significant fluctuations, with the highest extreme value recorded in 2006. Other notable peaks occur in 2003, 2007, 2011, 2015, 2017, and 2019, indicating that these years experienced particularly severe hydrological events in terms of human impact.

In contrast, the Health Infrastructure Loss Index is notably lower than human losses but shows sharp variations in certain specific years, such as 2012, 2019, and 2023. This less consistent and more sporadic behavior suggests that health infrastructure losses tend to be more isolated but potentially more severe in particular events. Furthermore, the mismatch in 2006 and 2015, years with high human loss indices but lower infrastructure damage, may indicate that the disasters primarily affected residential areas or social vulnerabilities rather than public health facilities.

**Figure 2:**
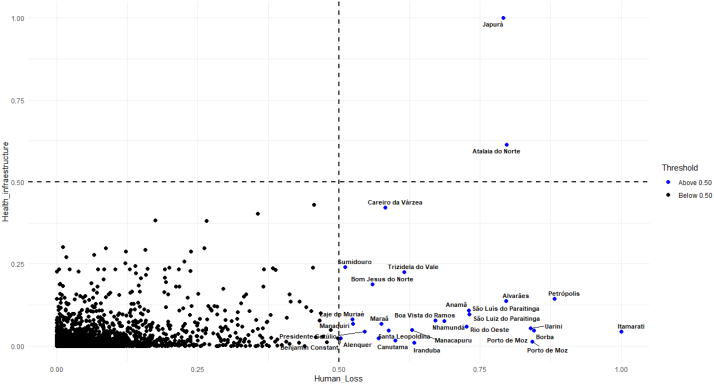
Municipal Distribution of Human Loss *versus* Health Infrastructure Loss with Threshold Classification (Brazil, 2000–2023).

Figure 2 presents a four-quadrant chart that classifies municipalities into four categories based on disaster characteristics using the HLI and HII: (i) High human losses and high health infrastructure losses; (ii) High human losses and low health infrastructure losses; (iii) Low human losses and high health infrastructure losses; and (iv) Low human losses and low health infrastructure losses.

Japurá and Atalaia do Norte, in the state of Amazonas, stand out as municipalities that require priority attention from public policies due to the high levels of human losses and damage to health infrastructure caused by hydrological disasters. These losses reflect failures in urban planning and management policies, indicating critical disruptions in health services, such as the loss of medical supplies and interruptions in care. Such impacts compromise emergency response and worsen the population's health conditions.

**Figure 3:**
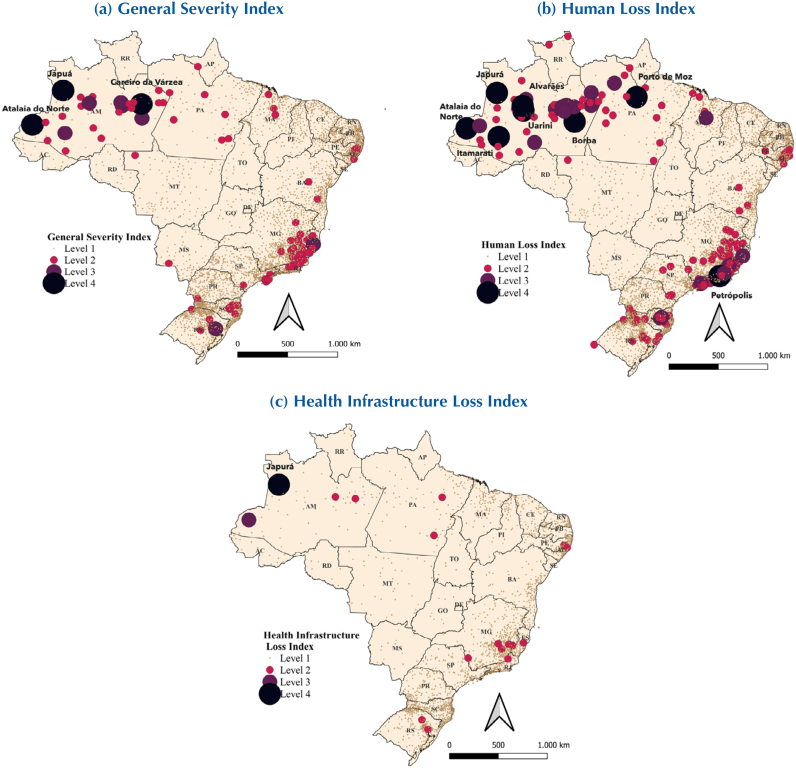
Spatial Distribution of General Severity, Human Loss, and Infrastructure Loss Indices by Municipality in Brazil (2000–2023).

Regarding the spatial distribution of the indices, high scores on the GSI (levels 4 and 3) are concentrated in the state of Amazonas (Figure 3). According to the Instituto Brasileiro de Geografia e Estatística (Brazilian Institute of Geography and Statistics) (2025), Japurá (1.00), Atalaia do Norte (0.82), and Careiro da Várzea (0.89) are small municipalities, with populations of 8,858, 15,314, and 19,637 in 2022, respectively. Similar to the GSI, losses in health infrastructure, as shown in Figure 3C, are especially significant in Japurá (1.00) and Atalaia do Norte (0.61), both in Amazonas.

On the other hand, the HLI (Figure 3B) is more widely distributed across the Brazilian territory. Losses are also evident in the northern Amazon region and along Brazil's densely populated southeastern coast. Among the municipalities in level 5, only Petrópolis is located in the Southeast and is the only large municipality, with a population exceeding 270 thousand.

**Figure 4:**
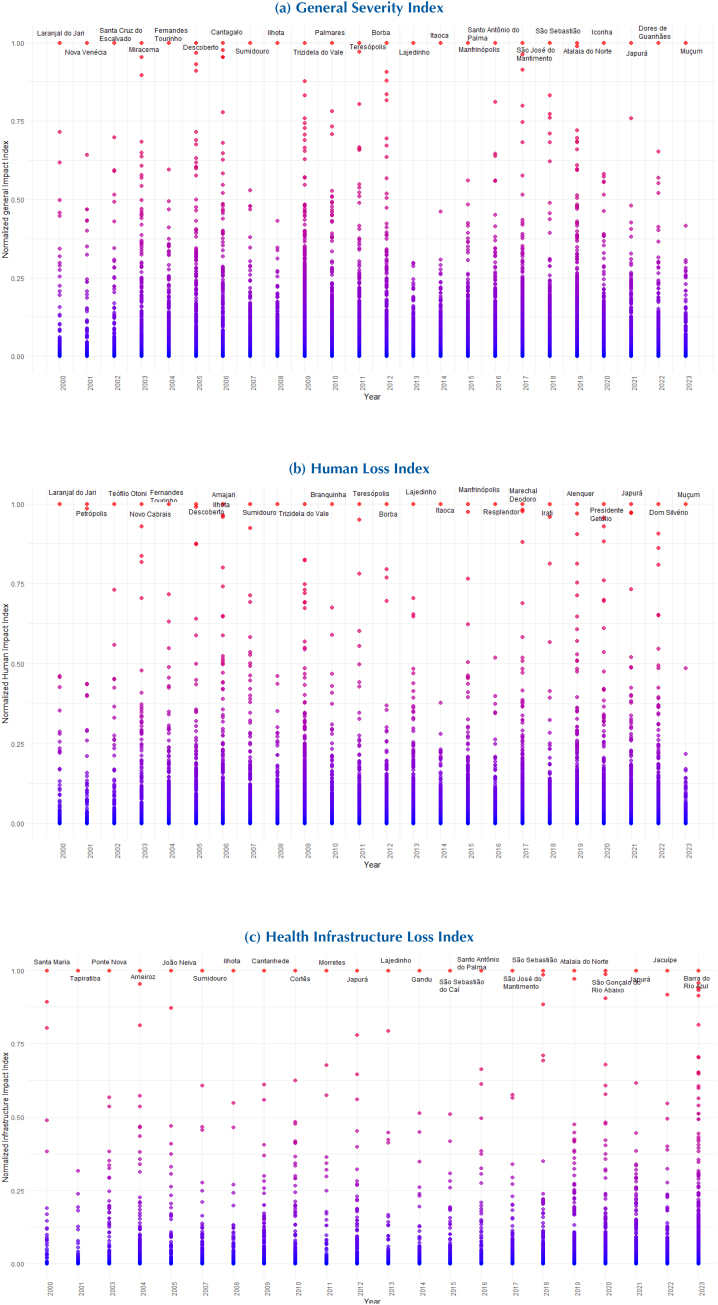
Temporal trends of the General Severity Index, Human Loss, and Health Infrastructure Loss by Municipality and Year in Brazil (2000–2023).

The annual distribution illustrated in Figure 4 provides further evidence of Brazil's most significant hydrological disasters between 2000 and 2023. In the North region, notable GSI occurrences affected even riverine and Indigenous communities along the Jari, Acre, Negro, and Amazon rivers, leading to major disasters in the municipalities of Laranjal do Jari (2000) in Amapá; Borba (2012), Atalaia do Norte (2019), and Japurá (2021) in Amazonas. In the Northeast region, the affected municipalities include small towns such as Trizidela do Vale (2009) in Maranhão, Palmares (2010) in Pernambuco, and Lajedinho (2013) in Bahia — with populations of 22,484, 54,584, and 3,527, respectively.

In the Southeast region, hydrological disasters occurred in all four states, namely: Nova Venécia (2001) and Iconha (2020) in Espírito Santo; Santa Cruz do Escalvado (2002), Fernandes Tourinho (2004), Descoberto (2005), Cantagalo (2006), São José do Mantimento (2017), and Dores de Guanhães (2022) in Minas Gerais; Miracema (2003), Sumidouro (2007), and Teresópolis (2011) in Rio de Janeiro; and Itaoca (2014) and São Sebastião (2018) in São Paulo. Finally, in the South region, noteworthy hydrological disasters occurred in Ilhota (2008) in Santa Catarina; Manfrinópolis (2015) in Paraná; and Santo Antônio do Palma (2016) and Muçum (2023) in Rio Grande do Sul — all municipalities with fewer than 20 thousand inhabitants^25^.

The results presented reveal the heterogeneity of the impacts of hydrological disasters in Brazil between 2000 and 2023, marked by disparities in human losses and damage to health infrastructure over time and across regions. The data confirm that such events disproportionately affect small, socioeconomically vulnerable municipalities, especially those in the North. Small municipalities, even those with master plans, often lack adequate infrastructure and risk management mechanisms^26^.

In the North region, the situation is worsened by the fact that health units are particularly fragile due to structural deficiencies, the absence of emergency protocols, and a shortage of human and material resources, leading to service disruptions during disasters^27^. The northern municipalities of Japurá, Atalaia do Norte, and Careiro da Várzea, for example, which reported high GSI scores, not only have high mortality rates but also face compounded challenges due to geographic isolation and predominantly Indigenous or mixed-race populations, which are considered more vulnerable to extreme events^25,28,29^. In these contexts, losses often exceed official records, leading to increased indirect mortality and morbidity^27^.

It is also important to highlight other Brazilian regions that experienced significant human losses and damage to health infrastructure. Although the Northeast is typically associated with droughts, there is a growing need to acknowledge the increasing incidence of hydrological disasters in the region. Due to low socioeconomic indicators and structural deficiencies, it is particularly exposed to public health crises during flood events^20^. Conversely, even in the South and Southeast regions, this does not guarantee adequate urban infrastructure or specific risk management legislation. Vulnerability is strongly tied to economic factors, public policies, and municipal size^26^.

Spatially, in the South, Southeast, and parts of the Northeast, hydrological disasters are predominantly characterized by sudden floods, often associated with intense rainfall and landslides triggered by saturated soils. These events tend to result in immediate fatalities and physical destruction of health infrastructure, contributing to high human loss values and, in some cases, elevated infrastructure damage. In contrast, in the North region, floods are typically gradual and extensive, with municipalities experiencing prolonged inundation that may affect large portions of their territory. In these contexts, health facilities are less frequently destroyed but are often partially or fully inoperable due to access constraints, equipment damage, supply interruptions, and workforce displacement. This dynamic helps explain the high severity levels observed in the General Severity Index in the North, despite differences in infrastructure damage profiles compared to other regions.

Finally, the findings confirm that the municipalities most severely affected are generally the least prepared to respond, reinforcing the need for integrated strategies that combine climate adaptation, poverty reduction, and investments in public health infrastructure. Addressing these challenges requires coordinated efforts across sectors and levels of government to reduce exposure, strengthen local capacity, and ensure that the most vulnerable populations are not left behind in disaster response and recovery processes.

## DISCUSSION

As climate change intensifies the frequency and severity of extreme events, the urgency for effective public policies aimed at risk mitigation and resilience-building increases. The findings of this study highlight the complexity of hydrological disasters in Brazil and their disproportionate impacts on human losses and health infrastructure. These results reinforce the need for decision-making based on systematized data and scientific evidence.

The analysis of human losses revealed a relatively stable trend over time, suggesting possible positive effects of policies already implemented. In contrast, damage to health infrastructure showed high variability, indicating vulnerabilities that have not yet been addressed systematically. The structural and operational vulnerability of health facilities in the face of hydrological disasters demands urgent investments in both physical and functional resilience.

In this context, territorial planning must be improved with a focus on regional equity, control of occupation in high-risk areas, and promotion of resilient infrastructure. Measures such as basic sanitation, urban drainage, and slope containment are crucial to reducing the exposure of vulnerable populations. At the same time, the integration of quantitative and qualitative data can guide more precise and effective public policies, especially when supported by environmental monitoring technologies and disaster forecasting systems.

In addition, financing policies should prioritize health infrastructure in the most affected areas, particularly in the North region. Specific credit lines and contingency plans for hydrological emergencies can significantly mitigate damage. Given the concentration of highly impacted municipalities, public consortia emerge as a viable alternative for regional cooperation in infrastructure and service projects.

There is also a pressing need for a National Climate Change Adaptation Policy aligned with regional strategies, considering Brazil's socio-environmental diversity. However, more than half of the state capitals have yet to complete their adaptation plans, and structural failures persist in disaster management, including institutional discontinuity, a lack of integration across government levels, and the predominance of reactive measures^30,31^.

The adaptive capacity of Brazilian cities is directly linked to political will, institutional structure, and strategic integration of the climate agenda^32^. Thus, rethinking disaster policy in Brazil requires a territorially integrated governance approach, guided by data and focused on reducing inequalities and strengthening long-term resilience.

## CONCLUSIONS

This study developed and applied composite indices to analyze immediate losses from hydrological disasters in Brazil (2000–2023), focusing on human losses and damage to health infrastructure. Disasters occurred mainly in small municipalities across all regions, typically with low institutional capacity and limited resources for effective risk management. Human losses were highest in the North, where vulnerable populations face extreme poverty, low physician density, poor access to potable water, and fragile infrastructure. Damage to health facilities, though more spatially dispersed, overlapped with areas of high human loss, reinforcing the coexistence and amplification of vulnerabilities.

Hydrological disasters in Brazil are mediated by social inequalities, deficient urban planning, and weak preventive policies. The prevailing reactive approach is costly and ineffective in reducing future losses. These findings underscore the need for territorially integrated policies that combine climate adaptation, socio-environmental justice, and institutional strengthening, particularly in the health sector. Future research should explore spatial spillover effects and broaden the index to other disaster types.

## Data Availability

The data are available upon request to the corresponding author.

## Footnotes

a Available from: https://www.undrr.org/implementing-sendai-framework/monitoring-sendai-framework
